# Engagement in Lifestyle Activities is Associated with Increased Alzheimer’s Disease-Associated Cortical Thickness and Cognitive Performance in Older Adults

**DOI:** 10.3390/jcm9051424

**Published:** 2020-05-11

**Authors:** Seongryu Bae, Sangyoon Lee, Kenji Harada, Keitaro Makino, Ippei Chiba, Osamu Katayama, Yohei Shinkai, Hyuntae Park, Hiroyuki Shimada

**Affiliations:** 1 Department of Preventive Gerontology, Center for Gerontology and Social Science, National Center for Geriatrics and Gerontology, 7-430 Morioka, Obu, Aichi 474-8511, Japan; sylee@ncgg.go.jp (S.L.); harada-k@ncgg.go.jp (K.H.); kmakino@ncgg.go.jp (K.M.); ichiba@ncgg.go.jp (I.C.); katayama.o@ncgg.go.jp (O.K.); yshinkai@ncgg.go.jp (Y.S.); shimada@ncgg.go.jp (H.S.); 2 Department of Health Care Science, Dong-A University, 37, 550-Gil Nakdongdaero, Saha, Busan 604-714, Korea; htpark@dau.ac.kr

**Keywords:** cortical thickness, cognitive function, physical activity, cognitive activity, social activity

## Abstract

The aim of this study was to examine the association between lifestyle activities, including physical, cognitive, and social activities, and Alzheimer’s disease (AD) signature cortical thickness, as well as to examine the mediating role of AD signature cortical thickness in lifestyle activities and cognitive function in community-dwelling healthy older adults. Participants were 1026 older adults who met the study inclusion criteria. The physical, cognitive, and social activities of daily life were assessed using a self-reporting questionnaire. AD signature cortical thickness was determined using FreeSurfer software. Cognitive function was evaluated using the National Center for Geriatrics and Gerontology-Functional Assessment Tool. Path analysis (based on structural equation modeling (SEM)) of cognitive activities indicated that the direct path from cognitive activities to cognitive function was significant (*p* < 0.001), as was the direct path from AD signature cortical thickness to cognitive function (*p* < 0.001). Physical (*p* < 0.05) or social activities (*p* < 0.05) had a direct effect on cognitive function. However, AD signature cortical thickness did not mediate the relationship between physical or social activities and cognitive function. Our findings suggest that higher levels of cognitive activities later in life have a significant and positive direct effect on cognitive function. Additionally, AD signature cortical thickness significantly mediates the relationship between cognitive activities and cognitive function.

## 1. Introduction

Aging is a major risk factor for neurodegenerative diseases such as Alzheimer’s disease (AD). As the proportion of the elderly population increases, the number of people with dementia increases dramatically with age, increasing healthcare costs and caregiver burden [1]. Therefore, it has become increasingly important to identify protective factors for delaying or decreasing cognitive decline. Daily multimodal activities that incorporate varying levels of engagement in physical, cognitive, and social activities have a meaningful impact on community-dwelling elderly people [2,3]. Engagement in these multimodal lifestyle activities, either simultaneously or sequentially, have shown promising benefits in terms of cognitive function [4] and brain health [5,6,7] in cognitively normal older adults.

Greater levels of engagement in physical, cognitive, and social activities, assessed by responses to self-report questionnaires, have frequently been associated with higher cognitive function scores in cognitively normal or mild cognitive impairment (MCI) [8,9,10,11,12]. Many studies have reported that lifestyle activities affect not only cognitive function, but also brain structure, including whole-brain and regional gray matter volumes. Some studies have shown that greater engagement in physical activity is associated with larger brain volume (especially in the frontal and hippocampal areas of healthy older adults) as well as MCI [13,14,15]. Additionally, interventional studies have suggested that physical activity may increase frontal and hippocampal volumes in community-dwelling healthy older adults [16,17]. Regarding cognitive activities, higher participation in cognitive leisure activities has been associated with better cognitive performance and reduced brain atrophy (e.g., in the middle frontal gyrus, hippocampus, anterior cingulate cortex, and posterior cingulate cortex) [18]. A more active cognitive lifestyle is associated with increased frontal and parietal brain volumes in healthy older adults [19]. Social activity is also closely related to greater normal-appearing white matter and gray matter volumes in healthy older adults [20,21]. Interventional studies have shown that social interaction groups experience significant increases in whole brain volume during the intervention period. As mentioned above, brain structure likely mediates the link between lifestyle activities and reduction in the risk of cognitive decline.

However, it remains unclear whether greater engagement in physical, cognitive, and social activities causes any changes in cortical thickness. The cortex is a tightly folded sheet of neurons, ranging in thickness from 1.5 to 4.5 mm in different cortical regions [22]. Cortical thickness is calculated as the mean distance from the grey–white matter boundary to the grey–cerebrospinal fluid boundary within a defined cortical region [23]. The gray matter is the location of the neuro bodies, whereas the cortical thickness can reflect the cytoarchitectural features of the neuropil, including the size, density, and arrangement of neurons, neuroglia, and nerve fibers, rather than the number of neurons. The measurement of cortical thickness allows more precise measurement in deep sulci and analysis of the morphology as a cortical sheet [24]. Measurements of cortical thickness may be more closely linked with cognitive ability than volumetric or intensity-based gray matter concentration measures, although this has been shown to relate to other local measures of gray matter [25]. Cortical thickness has been suggested as closely associated with cognitive functioning [26]. Verbal memory performance has been found to be associated with the medial temporal cortical thickness in normal subjects [27]. Slower Trail Making Test part B (TMT-B) completion times have been associated with cortical thinning in the frontal and temporal regions [28]. Another study suggested that the thickness of the salience network predicted executive functions and mediated the age–cognition relationship for executive function [29]. Furthermore, another study reported that a decrease in cortical thickness could be detected in cognitively normal individuals several years before the onset of clinical symptoms [30]. Therefore, measures of cortical thickness may provide important information about the integrity of areas of the cerebral cortex, which, in turn, may provide new insights into the relationship between lifestyle activities and cognitive function.

Several imaging biomarkers exist for detecting AD-related neuropathology. Positron emission tomography (PET) and cerebrospinal (CSF) biomarkers are the gold standard for identifying individuals with molecular evidence of AD neuropathology, but these procedures are invasive, expensive, and only accessible in specialized centers [31,32]. On the other hand, structural magnetic resonance imaging (MRI) is noninvasive, less expensive, and one of the most readily available methods obtained from morphometric estimates. Structural MRI studies examining AD signatures have focused on global atrophy [33,34], hippocampal volume atrophy [35,36], and an AD signature consisting of a composite of thickness estimates derived from regions impacted early during the course of AD [37,38]. AD signature cortical thickness, including cortical thinning in the inferior temporal lobes, middle temporal lobes, entorhinal cortex, and fusiform gyrus, has been used as a general indicator of AD-associated neurodegeneration [25]. It is associated with memory performance, cognitive decline, and progression to dementia [39,40,41,42,43,44], and is a better predictor of progression from mild cognitive impairment (MCI) to AD than hippocampal volume [45]. It is also closely associated with AD-like CSF characteristics [37]. AD signature cortical thinning is associated with increased regional amyloid-β deposition measured by PET [41]. Dickerson et al. (2012) found that changes in cortical thickness are detectable in cognitively normal adults who later develop AD dementia, and that individuals who express this marker have a three-fold increased risk for AD dementia over the ensuing decade [37]. Therefore, thinning in AD signature cortical thickness is a valid and reliable biological marker for AD. Although engagement in lifestyle activities has been associated with improved cognitive performance, the neural mechanisms underlying the protective effects of these lifestyle activities are largely unknown.

Thus, the aim of this study was to examine the associations between lifestyle activities (i.e., physical, cognitive, and social activities) and AD signature cortical thickness. In addition, we aimed to examine the mediating role of AD signature cortical thickness in relation to lifestyle activities and cognitive function in community-dwelling healthy older adults. We hypothesized that engagement in lifestyle activities may enhance AD signature cortical thickness, which in turn might mediate the relationship between lifestyle activities and cognitive performance.

## 2. Materials and Methods

### 2.1. Participants

Participants were recruited from a sub-cohort of the National Center for Geriatrics and Gerontology Study of Geriatric Syndrome (NCGG-SGS) [46]. This sub-cohort targeted older adults aged 60 years or more who lived in Takahama city, Aichi Prefecture, Japan. A total of 9716 individuals were invited to participate in the screening survey, which included an assessment of lifestyle activities and cognitive function, of which 4122 participated. Invitation letters for brain magnetic resonance imaging (MRI) assessment were sent to 4080 of these 4122 older adults, excluding those who had a pacemaker. In total, 1221 older adults participated in the MRI assessment. A total of 195 participants were excluded because of missing data (*n* = 39), a history of stroke (*n* = 62), Parkinson’s disease (*n* = 3), depression (*n* = 35), dementia (*n* = 1), other brain diseases (including brain tumor, cerebral infarction, chronic subdural hematoma) (*n* = 49), and failed processing during estimation of cortical thickness (*n* = 6). After excluding these individuals, we analyzed 1026 participants in this study. The study protocol was developed in accordance with the Declaration of Helsinki and was approved by the ethics committee of the National Center for Geriatrics and Gerontology (approval number: 861). Prior to study participation, informed consent was obtained from all participants.

### 2.2. Lifestyle Activities Questionnaire

The physical, cognitive, and social activities in daily life were assessed using a self-report questionnaire. The questionnaire consisted of three sections that covered physical, cognitive, and social activities and comprised 36 items. Physical activities items were selected based on the national sports life survey from the Sasakawa sports foundation [47], which predominantly demands physical fitness in older adults. Physical activity items included “walking”, “bicycling”, “jogging”, “swimming”, “strength training”, “yoga”, “gymnastics”, “dancing”, “hiking”, “playing golf”, “playing grand golf”, and “ball exercise”. We chose items for cognitive activities that had been rated as cognitively demanding in previous research [2,48,49,50] such as “writing a diary or letters”, “reading books”, “reading magazines or newspapers”, “learning activities”, “using a computer including internet use”, “doing crossword puzzles”, “playing board games (e.g., card games, Go or Japanese chess)”, “playing musical instruments”, “handicraft”, “listening to music”, “appreciate art”, and “make stocks and invest”. Items for social activities consisted of activities requiring social engagement and socializing with others in person, based on the Annual Report on the Aging Society, a national comprehensive social participation survey of older adults developed by the Japanese Cabinet Office [51], as well as a previous study [52]. The items included being “officer of a senior club or neighborhood association”, “attending a regional event”, “engage in environmental beautification activities”, “teaching activity”, “supporting activity”, “working”, “go to karaoke”, “eating out or tea party with friends”, “go shopping with friends”, “talk to friends (including phone)”, “attending an event or concerts”, and “go traveling”. Respondents reported the frequency of each activity over the prior 12 months as “never = 0”, “once a month or less = 1”, “several times a month = 2”, “1–2 times per week = 3”, “3–6 times per week = 4”, and “every day = 5”. The scores for each activity were summed to generate a physical activity score ranging from 0 to 60, a cognitive activity score ranging from 0 to 60, and a social activity score ranging from 0 to 60.

### 2.3. MRI Acquisition

Structural magnetic resonance imaging (MRI) was performed on a 3 T Siemens MAGNETOM Trio Tim 3T scanner (Siemens Medical Solutions, Erlangen, Germany) with a 12-channel head coil. A whole brain three-dimensional T1-weighted magnetization prepared rapid acquisition gradient echo (MPRAGE) sequence was acquired in the sagittal plane with the following parameters: repetition time (TR) = 1800 ms, echo time (TE) = 1.99 ms, flip angle = 9°, slices = 160, slice thickness = 1.1 mm, voxel = 1.0 × 1.0 × 1.1 mm, image matrix = 256 × 256 mm, and field of view (FOV) = 250 mm. Each scan took 4:06 min to complete.

### 2.4. Cortical Thickness Measurement

FreeSurfer version 6.0 was used to extract the surface area and cortical thickness. This image processing suite provides automated segmentation of both cortical and subcortical brain structures. Details of the technical procedures involved are documented elsewhere [53] and are freely available for download (http://surfer.nmr.mgh.harvard.edu/). Briefly, all images were automatically processed with skull stripping, spatial transforms, atlas registration, and spherical surface maps. After tessellating the grey–white boundary and locating the grey–pial boundary in FreeSurfer, cortical thickness was calculated as the closest distance between the grey–white matter boundary and the pial mesh at each vertex on the tessellated surface [23]. Automated cortical parcellation and region of interest (ROI) definition were performed using the Desikan–Killiany Atlas, which manually labelled 34 cortical ROIs in each hemisphere [54]. AD signature cortical thickness was defined by averaging cortical thickness of the entorhinal cortex, inferior temporal lobes, middle temporal lobes, and fusiform gyrus, a lower value indicating more severe AD pathology [25,55].

### 2.5. Cognitive Performance

Cognitive performance was assessed using the National Center for Geriatrics and Gerontology-Functional Assessment Tool (NCGG-FAT) [56]. Memory was measured using the Word List Memory and Story Memory tests. For the Word List Memory test, participants were instructed to memorize 10 words. Next, 30 words (the 10 target words and 20 distracters) were shown, and participants had to choose the 10 target words, repeated for three trials (i.e., immediate recognition). Participants were then instructed to recall (write down) the 10 target words after approximately 20 min (i.e., delayed recall). We calculated the composite score using the sum of the average of three trials of immediate recognition and delayed recall (range: 0 to 20). For the Story Memory test, participants were instructed to remember the details of a story, then immediately select the correct answer for 10 questions about the story (immediate recognition), then again after 20 min (delayed recognition).

We calculated the composite score using the sum of the immediate recognition and delayed recognition (range: 0 to 20). Attention and executive function were assessed using the Trail Making Test parts A and B (TMT-A and TMT-B). For TMT-A, participants were asked to touch the target numbers displayed randomly on the monitor in consecutive order (1–15). For TMT-B, participants were asked to touch target numbers and letters (Japanese Kana characters). They were asked to respond as quickly as possible. The TMT B-A score, calculated as the difference in times between TMT-B and TMT-A (i.e., TMT-B minus TMT-A), is considered a measure of cognitive flexibility relatively independent to manual dexterity [57]. Processing speed was assessed using the Symbol Digit Substitution Test (SDST), in which nine pairs of numbers and symbols were displayed at the top of the monitor. A target symbol was presented at the center of the monitor. Participants were asked to choose the number corresponding to the target symbol at the bottom of the monitor. The Mini-Mental State Examination (MMSE) was used to measure global cognitive function [58].

### 2.6. Statistical Analysis

The relationships among each variable (physical activity, cognitive activity, social activity, AD signature cortical thickness, and cognitive performance) were assessed using a partial correlation analysis by controlling for the covariates (age, sex, estimated total intracranial volume). Structural equation modelling (SEM) [59] was then performed to examine the mediating role of cortical thickness in relation to daily activity types and cognitive function. SEM was displayed as path diagrams, where the physical, cognitive, or social activities represent observed variables, respectively, and AD signature cortical thickness was shown as observed variables (square boxes). Cognitive performance (created from the Word List Memory and Story Memory tests, as well as MMSE, SDST, and TMT B-A scores) was represented as a latent variable (ovals). Single-headed arrows indicated standardized regression coefficients, and double-headed arrows indicated correlation coefficients. We measured some indices of model fit including chi-square values, the goodness of fit index (GFI), the comparative fit index (CFI), and the root mean square error of approximation (RMSEA) for SEM analysis. We considered a model an acceptable fit when it respected the following thresholds: low chi-square values relative to degrees of freedom with an insignificant *p*-value (*p* > 0.05); values > 0.95 for GFI and CFI; and values < 0.07 for RMSEA [60,61]. Data were analyzed using SPSS (version 25.0; SPSS Inc., IBM Corp., Chicago, IL, USA) and AMOS (version 25.0; SPSS Inc., IBM Corp., Chicago, IL, USA) software packages. Statistical significance was set at *p* < 0.05.

## 3. Results

### 3.1. Participants Characteristics

The demographic characteristics of participants are shown Table 1. The mean age was 70.6 years among the 1026 participants (524 men and 502 women). The average scores for physical, cognitive, and social activities were 8.5, 15.4, and 11.6, respectively, and average AD signature cortical thickness was 2.95 mm. In relation to cognitive function, the average scores for the Word List Memory test, Story Memory test, MMSE, and SDST were 11.5, 14.0, 27.7, and 46.9, respectively. Average TMT B-A performance time was 19.4 s.

### 3.2. Association of AD Signature Cortical Thickness and Cognitive Function with Lifestyle Activities

Table 2 shows the partial correlations among physical activity, cognitive activity, and social activity scores, AD signature cortical thickness, and cognitive performance. Cognitive activities were significantly correlated with AD signature cortical thickness, Word List Memory test score, Story Memory test score, MMSE score, SDST score, and TMT B-A. However, physical and social activities were not associated with AD signature cortical thickness. Additionally, physical activities were only significantly correlated with MMSE score, and for social activities, the only significant correlation was with SDST score. AD signature cortical thickness was associated with Word List Memory test score, Story Memory test score, and SDST score.

### 3.3. Structural Equation Model

**Figure 1:**
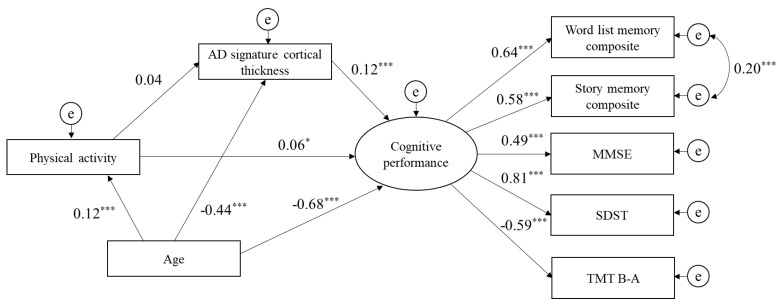
Structural model for associations of physical activity, AD signature cortical thickness, and cognitive performance. Goodness of fit index = 0.98, comparative fit index = 0.97, root mean square error of approximation = 0.06. e, error term; MMSE, Mini-Mental State Examination; SDST, Symbol Digit Substitution Test; TMT B-A, Trail Making Test part B minus part A. * *p* < 0.05, ** *p* < 0.01, *** *p* < 0.001.

**Figure 2:**
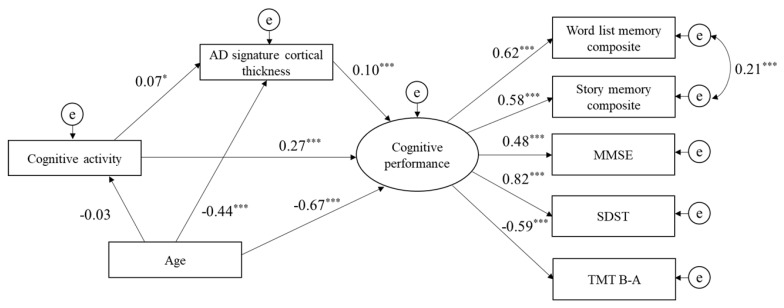
Structural model for associations of cognitive activity, AD signature cortical thickness, and cognitive performance. Goodness of fit index = 0.98, comparative fit index = 0.97, root mean square error of approximation = 0.06. e, error term; MMSE, Mini-Mental State Examination; SDST, Symbol Digit Substitution Test; TMT B-A, Trail Making Test part B minus part A. * *p* < 0.05, ** *p* < 0.01, *** *p* < 0.001.

**Figure 3:**
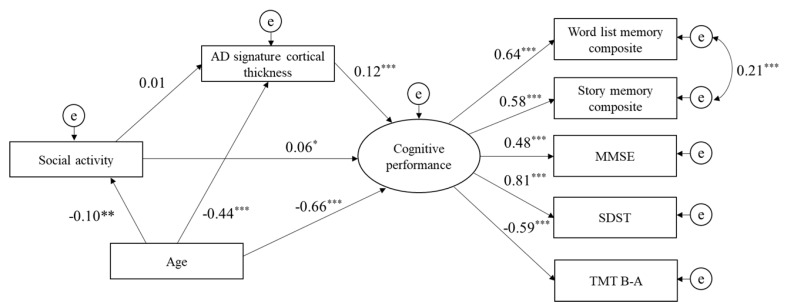
Structural model for associations of social activity, AD signature cortical thickness, and cognitive performance. Goodness of fit index = 0.98, comparative fit index = 0.97, root mean square error of approximation = 0.06. e, error term; MMSE, Mini-Mental State Examination; SDST, Symbol Digit Substitution Test; TMT B-A, Trail Making Test part B minus part A. * *p* < 0.05, ** *p* < 0.01, *** *p* < 0.001.

The path analysis based on SEM was tested separately for each of the three lifestyle activity types (physical, cognitive, and social). Physical activities had a direct effect on cognitive performance, and AD signature cortical thickness was significantly positively associated with cognitive performance. However, AD signature cortical thickness did not mediate the relationship between physical activities and cognitive performance. The indices of model fit were: chi-square = 82.4 (df = 16, *p* < 0.001), GFI = 0.98, CFI = 0.97, and RMSEA = 0.06 (Figure 1). The path analysis of cognitive activities indicated that the direct path from cognitive activities to cognitive performance was significant, as was the direct path from AD signature cortical thickness to cognitive performance. Additionally, AD signature cortical thickness significantly mediated the relationship between cognitive activities and cognitive performance. The indices of model fit were: chi-square = 89.5 (df = 16, *p* < 0.001), GFI = 0.98, CFI = 0.97, and RMSEA = 0.07 (Figure 2). Finally, the direct path from social activities to cognitive performance was also significant, as was the direct path from AD signature cortical thickness to cognitive performance. However, AD signature cortical thickness was not a mediator of the relationship between social activities and cognitive performance. The indices of model fit were: chi-square = 82.0 (df = 16, p < 0.001), GFI = 0.98, CFI = 0.97, and RMSEA = 0.06 (Figure 3).

## 4. Discussion

This study aimed to investigate the association between lifestyle activities (i.e., physical, cognitive, and social activities) and AD signature cortical thickness, and to examine the mediating role of AD signature cortical thickness in relation to lifestyle activities and cognitive performance. Our results indicate that cognitive activities were significantly correlated with AD signature cortical thickness, Word List Memory test score, Story Memory test score, MMSE score, SDST score, and TMT B-A. The findings from the current study were consistent with the results of another study that found a greater frequency of cognitive activities to be associated with enhanced cognitive performance. Cross-sectional studies have found that reading the newspaper is associated with stronger memory, fluid, and crystallized abilities [62], and that cognitive activity-related benefits may be found in perceptual speed among older adults [63]. A longitudinal study found that greater frequency of engagement in activities like reading and playing chess are associated with more gradual five-year declines in perceptual speed [64]. Willson et al. (2003) suggest that increased cognitive stimulation may reduce annual rates of global cognitive decline by up to 20% [65].

Our SEM showed that the direct path from cognitive activities to cognitive performance was significant, as was the direct path from AD signature cortical thickness to cognitive performance. Additionally, AD signature cortical thickness significantly mediated the relationship between cognitive activities and cognitive performance. These have important implications for healthy aging, as AD signature cortical thickness (comprising the inferior and middle temporal lobes, entorhinal cortex, and fusiform gyrus) is integral to memory function, and atrophy in these regions is an early biomarker of mild AD [25]. Busovaca et al. (2016) found AD signature cortical thickness to be associated with memory performance in younger and healthy older adults similarly, and that age group differences in memory performance were mediated by regions of AD signature cortical thickness [40]. Moreover, asymptomatic individuals with fibrillar forms of amyloid have cortical thinning in regions of AD signature cortical thickness compared with individuals without evidence of amyloid, suggesting that the degree of cortical thinning in regions of AD signature cortical thickness predicts progression to clinical AD among cognitively healthy older adults [39,41,42]. These findings indicate that thinning in AD signature cortical thickness is a valid and reliable biological marker for AD. Therefore, our findings suggest that the maintenance of AD signature cortical thickness through cognitive activities in older adults has important implications for cognitive function and dementia prevention.

Some randomized controlled trials have shown that cognitive training can improve cognition function and cortical thickness in older adults. Engvig et al. (2010) reported that eight weeks of memory training improved source memory performance. Memory training groups also showed increases in cortical thickness in the right fusiform and lateral orbitofrontal cortex compared with control groups [66]. Compared to single-domain cognitive training, multi-domain cognitive has training demonstrated more advantages in visuospatial/constructional, attention, delayed memory abilities, and left supramarginal and left frontal pole regions [67]. According to volumetric evidence, it has been shown that a more active cognitive lifestyle is related to greater frontal and parietal brain volume in healthy older adults [19]. Schultz et al. (2015) reported that higher cognitive leisure activity scores were associated with greater gray matter volumes in the middle frontal gyrus, hippocampus, and the anterior as well as posterior cingulate [18].

Although engagement in cognitive activities has been associated with better cognitive performance and increasing brain volumes in older adults, the neural mechanisms mediating the protective effects of these cognitive activities are largely unknown. One possible explanation for the better cognitive performance associated with an active cognitive lifestyle might be the existence of use-dependent plasticity of synaptic strength and brain structure [68,69]. Higher engagement in cognitive activities among older adults can provide stimulation for the cognitive system. Furthermore, according to the hypothesis of cognitive reserve, the enriched environment can influence neural processing and synaptic organization through cognitive activities by allowing neurological processes to become more efficient and adaptive [70].

In this study, although physical or social activities showed an association with cognitive performance, they were not associated with AD signature cortical thickness. Many previous studies have reported that physical activity is associated with greater gray matter volume [13,17,20]. There are several underlying putative mechanisms, including increased blood flow and angiogenesis, the induction of neurotrophic factors, and activation of the immune system. However, there have been few neuroimaging studies investigating the relationship between physical activity and cortical thickness. Lee et al. (2016) measured self-reported physical activity and cortical thickness in healthy older adults. They found longer durations of physical activity (≥1 hr/day), but not intensity or frequency, were associated with increased cortical thickness in the bilateral dorsolateral prefrontal, precuneus, left postcentral, and inferior parietal regions [71]. Another self-reported study reported that higher physical activity levels, operationalized as total MET minutes per week, were associated with increased cortical thickness in the right prefrontal cortex [72]. However, the questionnaire used in our study asked participants to indicate their frequency in performing various types of physical activity, but the duration or intensity of physical activity was not considered. As such, it is likely that the lack of association is explained by the self-reporting nature of the physical activity measurements.

Greater engagement in social activities has been associated with greater normal-appearing white matter and gray matter volumes [20,21]. However, to our knowledge, no previous studies have examined the relationship between social activity and cortical thickness in community-dwelling older adults. James et al. (2012) reported that higher social engagement was associated with greater total brain volume and greater temporal and occipital gray matter volumes [21]. They used an eight-item measure of social engagement that included questions more related to physical than social activities, such as hiking, bowling, working out, swimming, fishing, or golfing. Physical activity is more strongly associated with gray matter volume than social activity, and may have strongly influenced the responses to this question. On the other hand, Seider et al. (2016) found no association between social activities like interpersonal interaction and gray matter volume [73]. Previous studies examining the relationship between brain volume and social activity have been equivocal. The association between different types of social activity and cortical thickness data warrants further study. Nevertheless, considering prior research examining the relationship between brain volume and social activity, and the results of the current study, we cannot support our hypothesis that social activity is associated with greater AD signature cortical thickness.

Some limitations should be considered when interpreting the results. First, given that we used self-reported lifestyle activities in the questionnaire, recall bias is inherent. In addition, our lifestyle activities questionnaire was susceptible to a selection bias that may have led to underreporting of physical, cognitive, and social activities as it was created based on previous research and a national survey of older age adults. Second, since our study was designed to be cross-sectional, we cannot claim a causal relationship between lifestyle activities and cortical thickness. Lastly, selection bias should be considered another limitation. Healthy elderly might have participated in this study because they were able to participate in the screening survey at the community center. This corresponds to 42.4% of the target population. Furthermore, of the 4122 people who participated in the screening survey, 1221 older adults visited the laboratory to complete the MRI assessment (response rate: 29.6%). These limitations related to participants might have influenced the results of the present study. Nevertheless, the strengths of this study include the large sample size and the use of a combined concern of the three key individual lifestyle activity factors (i.e., physical, cognitive, and social) within an individual. However, it is noteworthy that this is the first study showing the role of engagement of lifestyle activities in age-related cortical thinning.

## 5. Conclusions

Our findings suggest that a higher level of cognitive activities later in life has a significant and positive direct effect on cognitive function. In other words, a higher cognitive function could also lead people to be more engaged in cognitively demanding activities. In addition, AD signature cortical thickness significantly mediated the relationship between cognitive activities and cognitive function. Further studies are necessary to confirm a causal association between lifestyle activities, cortical thickness, and cognitive function, using longitudinal data.

## Figures and Tables

**Table 1:** Baseline characteristics of study participants.

Characteristic	Mean or % (SD)	Range
Age, years	70.6 (6.3)	60 to 95
Sex, male	51.1	
Physical activity, score	8.5 (6.2)	0 to 34
Cognitive activity, score	15.4 (7.7)	0 to 46
Social activity, score	11.6 (6.4)	0 to 41
AD signature cortical thickness, mm	2.95 (0.13)	2.29 to 3.33
Entorhinal, mm	3.47 (0.28)	2.06 to 4.30
Inferior temporal, mm	2.81 (0.12)	2.22 to 3.25
Middle temporal, mm	2.78 (0.12)	2.34 to 3.21
Fusiform, mm	2.72 (0.13)	2.30 to 3.10
Word List Memory test composite, score	11.5 (3.1)	1.3 to 20.0
Story Memory test composite, score	14.0 (3.6)	0 to 20
MMSE, score	27.7 (2.2)	16 to 30
SDST, score	46.9 (9.7)	8 to 78
TMT B-A, second	19.4 (17.3)	−9 to 186

SD, standard deviation; AD, Alzheimer’s disease; MMSE, Mini-Mental State Examination; SDST, Symbol Digit Substitution Test; TMT B-A, Trail Making Test part B minus part A.

**Table 2:** Partial correlations among activities, cortical thickness, and cognitive function.

	Partial Correlation Coefficient							
	1	2	3	4	5	6	7	8
1. Physical activity, score								
2. Cognitive activity, score	0.187 ***							
3. Social activity, score	0.218 ***	0.304 ***						
4. AD signature cortical thickness, mm	0.048	0.079 **	0.009					
5. Word List Memory test composite, score	0.058	0.156 ***	0.051	0.15 ***				
6. Story Memory test composite, score	0.035	0.209 ***	0.021	0.07 *	0.382 ***			
7. MMSE, score	0.087 **	0.146 ***	−0.009	0.051	0.289 ***	0.28 ***		
8. SDST, score	0.037	0.28 ***	0.069 *	0.11 ***	0.345 ***	0.239 ***	0.251 ***	
9. TMT B-A, second	−0.044	−0.182 ***	−0.044	−0.025	−0.297 ***	−0.226 ***	−0.265 ***	−0.313 ***

AD, Alzheimer’s disease; MMSE, Mini-Mental State Examination; SDST, Symbol Digit Substitution Test; TMT B-A, Trail Making Test part B minus part A. * *p* < 0.05, ** *p* < 0.01, *** *p* < 0.001.
